# Non-Invasive Blood Pressure Tracking of Spontaneous Hypertension Rats Using an Electronic Nose

**DOI:** 10.3390/s24010238

**Published:** 2023-12-31

**Authors:** Fumei Zhang, Lijing Yang, Jia Wei, Xiaojing Tian

**Affiliations:** 1 China-Malaysia National Joint Laboratory, Biomedical Research Center, Northwest Minzu University, Lanzhou 730030, China; 278061349@xbmu.edu.cn (F.Z.); y220830418@stu.xbmu.edu.cn (L.Y.); 284142345@xbmu.edu.cn (J.W.); 2 Department of Medicine, Northwest Minzu University, Lanzhou 730124, China; 3 School of Life Sciences and Engineering, Northwest Minzu University, Lanzhou 730124, China; 4 Gannan Yak Milk Research Institute, Gannan 747000, China

**Keywords:** spontaneous hypertension rat, fecal odor, electronic nose, non-invasive, blood pressure

## Abstract

Traditional noninvasive blood pressure measurement methods in experimental animals are time consuming and difficult to operate, particularly for large numbers of animals. In this study, the possibility of sensing fecal odor to estimate the blood pressure status of spontaneous hypertension rats (SHRs) was explored with the aim of establishing a new method for non-invasive monitoring of blood pressure. The body weight and blood pressure of SHRs kept increasing with growth, and the odor information monitored using an E-nose varied with the blood pressure status, particularly for sensors S6 and S7. The fecal information was analyzed using principal component analysis, canonical discriminant analysis and multilayer perception neural networks (MLP) to discriminate SHRs from normal ones, with a 100% correct classification rate. For better prediction of blood pressure, the model built using multiple linear regression analysis, partial least squares regression analysis and multilayer perceptron neural network analysis were used, with coefficients of determination (*R*^2^) ranging from 0.8036 to 0.9926. Moreover, the best prediction model for blood pressure was established using MLP analysis with an *R*^2^¬ higher than 0.91. Thus, changes in blood pressure levels can be tracked non-invasively, and normotension can be distinguished from hypertension or even at different hypertension levels based on the odor information of rat feces, providing a foundation for non-invasive health monitoring. This work might provide potential instructions for functional food research aimed at lowering blood pressure.

## 1. Introduction

As a leading cause of death and disability, hypertension remarkably increases the risk of developing heart disease, stroke, kidney disease, and other diseases [1]. Therefore, monitoring the blood pressure and adopting antihypertensive therapy are necessary to effectively inhibit the occurrence of cardiovascular diseases and their complications and reduce the absolute risk of cardiovascular death [2]. Animal models, particularly for spontaneous hypertension rats (SHRs), were used to study the pathogenesis, treatment and prevention of hypertension [3,4,5]. At present, the blood pressure level of humans can be easily detected at home or at a medical point. However, some challenges in animal blood pressure detection are still encountered.

Methods for animal blood pressure monitoring were divided into invasive and non-invasive methods [6]. The invasive method, including implantable telemetry and femoral artery cannulation, could directly obtain intravascular pressure. Both methods require the anaesthetization of animals and burying the sphygmomanometer probe into their peripheral artery to measure arterial pressure [7], posing risks of infection, and both processes are technically challenging and complex [8]. Moreover, invasive methods are not suitable for blood pressure testing for large numbers of animals. In reducing the risks of invasive blood pressure testing and improving safety and efficiency, non-invasive methods were developed. Animal blood pressure values were obtained by analyzing relevant feature signals, such as pulse wave, electrocardiography, arterial vibration, sound frequency, and arterial tension, indirectly reflecting blood pressure through blood flow, which are relatively easier to perform. Doppler sphygmomanometry [9] and oscillometry are the most commonly used non-invasive methods, placing a cuff on the animal’s tail or limb [10]. However, these traditional methods are time consuming because the movements of experimental animals such as rats are easily stimulated by the operation; moreover, the prolonged use may cause nerve and skin damage to rats [11,12,13]. Portable methods based on infrared sensors have been developed to detect changes in blood flow waves [14,15], but the systems are complex and not applicable to all testing animals. Hence, establishing a fast, non-invasive blood pressure measurement method for animals, particularly for a large number of animals, is necessary.

Volatile organic compounds (VOCs) released from the metabolites of microbial activity can be used to diagnose diseases [16]. The patterns of VOCs and biomarker signatures for a variety of non-communicable diseases have more applications in animal disease diagnosis by monitoring of urine [17], feces [18], blood, exhaled breath, sweat, and saliva using GC-MS, GC-IMS and E-nose. The E-nose has been used to diagnose tuberculosis [19,20], avian influenza [21], and wild boar mycobacteriosis caused by Mycobacterium bovis using exhaled gas and fecal samples [22,23]. Using exhaled breath, the period of developing acute liver failure and the healthy period in control rats were classified accurately using an E-nose [24]. Using VOCs in urine, the urine of patients with prostate cancer and that of healthy controls were distinguished using an E-nose [25]. Previous studies have found that hypertension affects the richness and diversity of intestinal flora, with an increased number of lactate-producing Bacteria Proteobacteria, altered ratio of Firmicutes/Bacteroidetes (F/B), and decreased number of Actinomycetes, Bifidobacteria, Bacteroides [26,27] and short-chain fatty acid (SCFA)-producing bacteria [28]. Metabolite changes were driven by the intestinal flora of hypertensive people, with a change in the level of fecal SCFAs [29,30], trimethylamine oxide [31,32], corticosterone [33], and content of indole acetic acid. Moreover, fecal odor changes were due to changes in intestinal flora and its metabolites, making it possible for non-invasive monitoring of animal blood pressure changes based on odor information.

In the present work, changes in blood pressure, body weight of SHRs and fecal odor of SHRs were monitored during breeding using an E-nose. This study primarily aimed to investigate the change trend of fecal odor with the increase in the blood pressure of SHRs, explore the relationship between fecal odor and blood pressure, predict the blood pressure status of SHRs using fecal odor, and establish a fast and non-invasive method for the detection of blood pressure based on odor information of gut microbe metabolites.

## 2. Material and Methods

### 2.1. Hypertensive Animal Model

A total of 24 5-week-old spontaneous hypertension rats (SHRs) were provided by the Experimental Centre of Lanzhou University, China, with 12 females and 12 males. Each SHR was housed in a separate cage in a clean environment with clear days and nights for one week to acclimate to the new surroundings. The animal experimentation in this study was approved by the Committee of Laboratory Animal Welfare and Ethics of Northwest Minzu University. After 1 week of acclimatization, the SHRs were fed for 12 weeks.

### 2.2. Test Methodology

#### 2.2.1. Weight and Blood Pressure Determination

During 12 weeks of breeding, the systolic blood pressure (SBP) and diastolic blood pressure (DBP) of SHRs were measured using a non-invasive blood pressure monitor (Tymon Non-invasive Blood Pressure Monitor (BP-100A), Chengdu Tymon Software Co., Ltd., Chengdu, China) every 3 weeks (6th, 9th, 12th, 15th, and 18th weeks). The body weight was monitored simultaneously, with 5 technical repetitions. The mean and standard deviation (X ± s) of blood pressure was used calculated.

#### 2.2.2. Collection of Fecal Samples

A total of 40 pellets of female and male rat feces were collected every 3 weeks. Then, the collected pellets were placed in 5 mL sterile centrifuge tubes and stored at −80 °C until the detection of odor information.

#### 2.2.3. Odor Information Collection

The odor information of fecal samples was obtained using a PEN3 portable E-nose (AIRSENSE, Schwerin, Germany) consisting of a sensor array of 10 metal oxide gas sensors [19], sampling and cleaning channels, a data acquisition system and a computer. Before the test, the samples were brought back to room temperature and the odor information was collected according to the method described by Ming Long et al. [34] with modification. The experimental conditions for the E-nose were as follows: 1 pellet of rat feces was placed in a beaker of 150 mL at room temperature and sealed with plastic wrap for a headspace generation time of 10 min. Before one sample was detected, the sensors were cleaned with fresh dry air for 80 s, to prevent the influence of residual odor from the previous sample. The sensors were then exposed to sample volatiles and the changes in the sensor responses were recorded by the data acquisition system of the E-nose. During the sampling process, the sample gas was transferred into the sensor chamber at a flow rate of 200 mL min^−1^, and the collection time was 80 s at an interval of 1 s. For each group, 40 repetitions were detected, and the first 1–3 repetitions were eliminated as they were adapting to the odor of feces.

### 2.3. Statistics

Multiple comparisons were used to investigate the differences in the sensor response of fecal samples of SHRs with different blood pressure status. Principal component analysis (PCA) and canonical discriminant analysis (CDA) were used to differentiate SHRs with normotension and hypertension. Multiple linear regression (MLR), partial least squares regression (PLS) and multilayer perception neural networks (MLP) were used to investigate the relationship between fecal odor information and blood pressure of SHRs. PCA and CDA were performed using SAS V9 for multiple comparisons, MLR and MLP analyses were performed using SPSS 25, and PLS was performed using Minitab 19. The results were plotted using Origin 2021.

## 3. Results

### 3.1. Blood Pressure and Body Weight of SHRs

**Figure 1:**
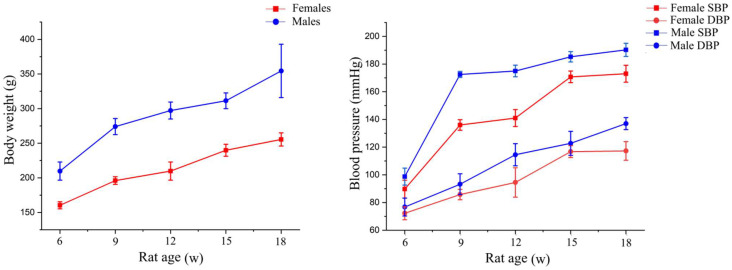
Changes in blood pressure and body weight of SHRs.

The body weight and blood pressure of SHRs are shown in Figure 1. The blood pressures of SHRs were all within the normotensive range (SBP115 [35]) in the 6th week and exceeded the normotensive range in the 9th week. In accordance with the 2017 ACC/AHA Hypertension Guideline and Clinical Practice Guide for Hypertension (Table 1) in China [35,36], the blood pressure of female SHRs reached Stage 1 hypertension in the 9th week and Stage 2 hypertension in the 12th week, and it continued to rise during breeding. The blood pressure of male SHRs reached Stage 2 hypertension in the 9th week. During the whole breeding period, the SBP, DBP and body weight of SHRs kept increasing. Furthermore, SBP and DBP of male SHRs were higher than those of female SHRs.

### 3.2. Characteristic Response of E-Nose to Fecal Samples of SHRs

**Figure 2:**
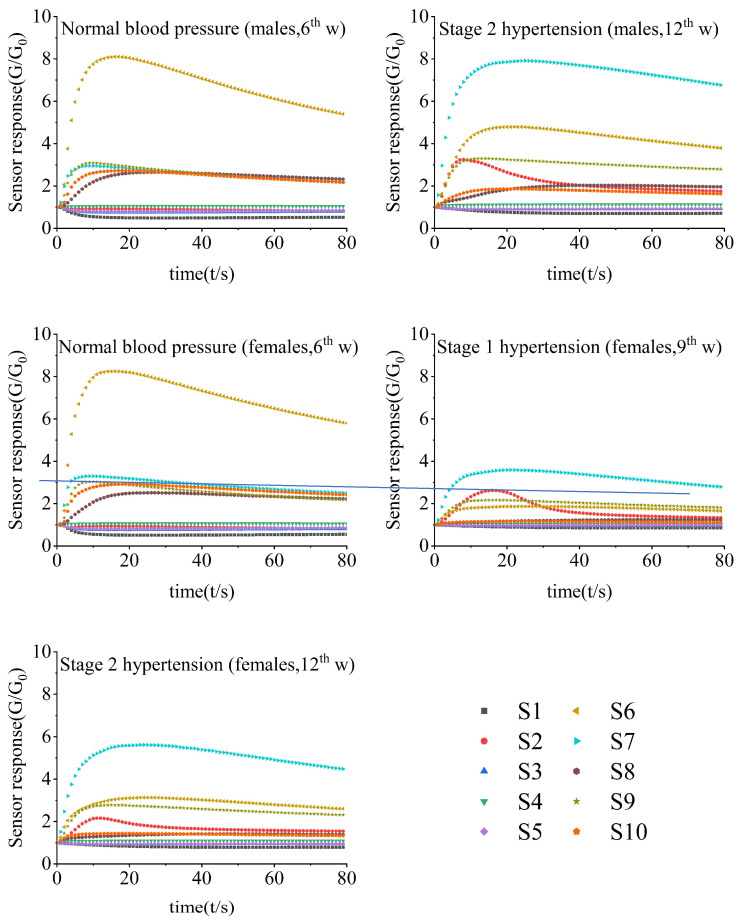
Characteristic response of E-nose to SHR fecal samples.

Figure 2 showed the characteristic response curves of the E-nose for male and SHRs with different blood pressures. Once contacting with the volatile compounds in fecal samples, the sensors quickly responded, especially for sensors S6–S10. After 60 s of detection, the sensor responses equilibrated gradually, and the final responses at 80 s were extracted for further analysis.

The signals corresponding to female SHRs with normal blood pressure greatly differed from those of hypertensive ones. Take the responses of sensor S6, for example: the G/G_0_ expanded to more than 8 in 12 s for SHRs with normal blood pressure, much greater than that of hypertensive ones, while the responses of sensors S7 and S9 were greater than those of normotensive ones.

### 3.3. Effect of Blood Pressure on Fecal Odor Information of SHRs

To investigate the effect of blood pressure on fecal odor, multiple comparisons with least significant difference were conducted using SPSS on the extracted data set; the results are shown in Table 2.

With the increase in blood pressure of male SHRs, the responses of sensors S6, S8, S9, and S10 decreased from the 6th to 12th week and then increased from the 12th to 18th week, whereas the responses of sensors S1, S2, S3 and S5 showed the opposite trend. The responses of sensors S4 and S7 continuously increased during the whole process.

The fecal odor response of female SHRs was consistent with that of male ones. The response of sensors S6, S8, S9 and S10 decreased from the 6th to 9th week and then increased from the 9th to 18th week. The responses of sensors S1, S3, and S5 increased from the 6th to 9th week and then remained stable. The response of sensor S2 increased from the 12th to 18th week. The response of sensors S4 and S7 continuously increased during the whole process.

**Figure 3:**
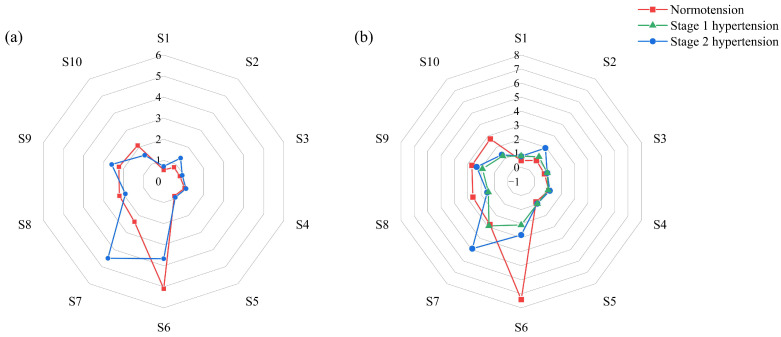
Radar plot of fecal odor from SHRs with varied blood pressure level ((**a**) male SHRs, (**b**) female SHRs).

In distinguishing the difference in fecal odor of SHRs with varied blood pressure, the final responses at 80 s were given in the form of a radar plot. As shown in Figure 3 and Table 2, the responses of sensors S6, S8, and S10 to SHRs with normotension were higher than that of SHRs with hypertension, while the responses of sensors S1, S2, S3, and S7 showed an opposite trend. In Figure 3, for SHRs with hypertension, Stage 1 or Stage 2 showed a similar response pattern, although Stage 1 had lower values. For SHRs with normotension, both genders shared a similar response pattern.

### 3.4. Qualitative Discrimination of SHRs with Different Blood Pressure Levels

**Figure 4:**
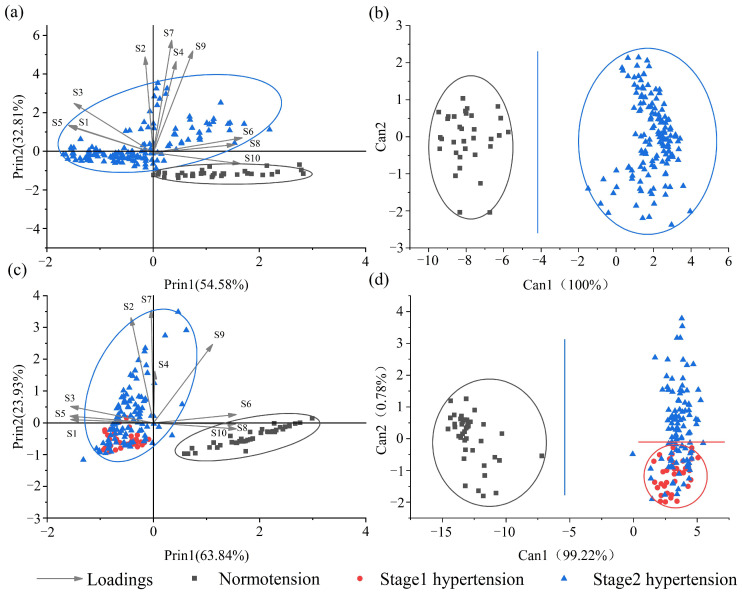
PCA and CDA results of E-nose signals for fecal samples from SHRs with different blood pressure levels ((**a**) male PCA, (**b**) male CDA, (**c**) female PCA, (**d**) female CDA).

PCA, CDA and MLP analysis were applied to build a model to estimate the blood pressure level using fecal odor information. The results are shown in Figure 4 and Table 3 and Table 4.

As shown in Figure 4a,b, the PCA results of male SHRs showed that the first principal component (Prin1) accounted for 54.58% and the second principal component (Prin2) accounted for 32.81%, revealing a total of 87.39% of the original information. SHRs with Stage 2 hypertension were discriminated from the normal ones, with no overlap of data points. Hypertensive SHRs were located at the upper part of the plot. Using form loading analysis, according to prin2, sensors S2, S4, S7, and S9 contributed to the distinguishing of hypertension SHRs. And in the direction of prin1, S6, S8, and S10 contributed to the discrimination of the normotension group, and S1, S3, and S5 contributed to distinguishing the hypertension group. CDA analysis showed a better discrimination effect, with hypertensive SHRs scattered in the area of Can1–2 and normotensive rats scattered in the area of Can1–5.

As shown in Figure 4c,d, the PCA results for female SHRs showed that Prin1 accounted for 64.84% and Prin2 accounted for 23.93%, which reveals a total of 88.77% of the original information. SHRs with hypertension were distributed in the left part of the plot with Prin1 < 0.5, away from the normal ones, although scatters of Stage 1 and Stage 2 hypertension overlapped with each other. Using form loading analysis, according to prin1, sensor S6, S8, and S10 contributed to the distinguishing of normotension SHRs, while S1, S3, and S5 contributed to the discrimination of the hypertension ones. According to prin2, sensor S2, S4, S7, and S9 contributed to an increase in blood pressure for the hypertension group. In CDA analysis, Can1 (99.22%) and Can2 (0.78%) explained 100% of the original information. Thus, better discrimination results were obtained, with Can1 = −5 for the separation of normotensive SHRs and hypertensive ones. For the discrimination of Stage 1 and Stage 2 hypertension, Stage 1 was located in the lower part with Can2 < 0, and fewer overlaps were observed.

Therefore, the blood pressure status could be distinguished on the basis of fecal odor information in male and female SHRs using PCA and CDA. However, the separation of Stage 1 and Stage 2 hypertension groups needs further study.

A MLP model with a 10–8–4 grid structure was built to estimate the blood pressure status. The number of input neurons of the MLP was 10, and the input information was obtained from fecal odor signals obtained using E-nose sensors. In addition, eight neurons were selected in the hidden layer. The output layer had four neurons, representing the blood pressure level of both genders. As shown in Table 4, for the classification of normotension and hypertension, the accuracy of the MLP neural network analysis for female and male SHRs was 95.36%, 76.02%, 65.94%, and 83.65%. The grid confusion matrix created indicated that the accuracy, precision, sensitivity, specificity and area under the curve of the blood pressure status discrimination area were higher than 65.94%, 54.55%, 19.42%, 75.56% and 56.86%, respectively (Table 4).

Regardless of gender, MLP with a 10–6–2 grid structure was used to estimate the blood pressure level, with ten E-nose responses as input, six neurons in the hidden layer, and two states of normotension and hypertension in the output layer. As shown in Table 3, the classification accuracy was increased to 100%, and the established grid confusion matrix indicated that the accuracy, precision, sensitivity, specificity and AUC for blood pressure state discrimination were improved to 100% (Table 4). In combination with the MLP neural network, qualitative discrimination between normotension and hypertension could be achieved with the E-nose sensor response signal.

When distinguishing between genders, the correct recognition rate is low, which may be due to the mixing of Stage 1 and Stage 2 hypertension in females as hypertension.

### 3.5. Quantitative Predictive Model of Blood Pressure

**Figure 5:**
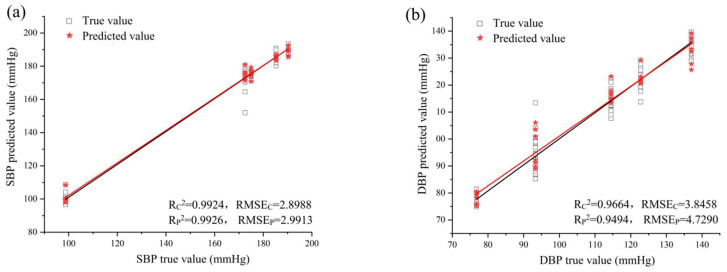
Quantitative prediction of blood pressure in male SHRs using MLP ((**a**) SBP, (**b**) DBP).

To establish the relationship between the blood pressure of SHRs and fecal odor signals obtained using an E-nose, MLR, PLS and MLP analyses were conducted. The feces of SHRs were randomly divided into a modelling set (30510) and validation set (75). The predictive effects of the three methods were compared to find the best prediction model, using the correlation coefficient (*R*^2^) and root mean square error (RMSE) between the predicted and experimental values, as larger *R*^2^ and lower RMSE leads to better calibration models. The results are shown in Table 5 and Figure 5.

#### 3.5.1. Male SHRs 

MLR, PLS and MLP were proven to be effective in predicting the SBP of *male SHRs* with coefficients of determination (*R_C_*^2^) higher than 0.93 and RMSE_C_ values lower than 8.24 in the training set. When the models were used to predict SBP, R_P_^2^ was above 0.95, and RMSE_P_ was below 7.42. In the predictive model of DBP, the coefficients of determination (0.92) were slightly lower than that of SBP, whereas the RMSE values were improved in the training and testing data set.

For predicting SBP and DBP, MLP analysis had the highest coefficient of determination and lower RMES values (*R*^2^ ≥ 0.9494 and RMSE ≤ 4.7290), as well as the best modelling and prediction among the three methods (Table 5). Visualizing the predicted effects of SBP and DBP established using MLP, the relationship between true and predicted values is plotted in Figure 5.

#### 3.5.2. Female SHRs 

In the predictive model built for the SBP of *female SHRs* using MLR, PLS and MLP, the coefficients of determination *R_C_*^2^ were higher than 0.92 and the RMSE_C_ was lower than 9.5 in the training set. In the testing set, the coefficients of determination were higher than 0.88. However, the coefficients of determination in the predictive model of DBP were lower than the former models, with an *R_C_*^2^ higher than 0.83.

Similar to male ones, MLP had the best performance for the prediction of blood pressure in female SHRs.

#### 3.5.3. No Distinction of Gender

The three methods were applied to the combined data set of male and female SHRs, and the predictive results were not improved, with an *R_C_*^2^ higher than 0.82 and RMSE_C_ lower than 14.21 for SBP in the training set, and an *R_C_*^2^ higher than 0.79 and RMSE_C_ lower than 10.12 for DBP in the training set. However, the models developed using MLP had high coefficients of determination and low RMSEs (*R*^2^ ≥ 0.9161 and RMSE ≤ 9.8250), and such models were considered as the most effective in modelling and prediction (Table 5).

For the prediction of DBP using fecal odor, most of the RMSE values were lower than 8 mmHg, regardless of the methods used and gender influence.

Therefore, the excellent prediction achieved using an E-nose was found by combining PLS, MLR and MLP for prediction of the blood pressure level for SHRs with *R*^2^ values higher than 0.79 and RMSE values lower than 14.21. The best results were found in the MLP model, with the highest correlation (*R*^2^ = 0.99) in the training set. MLP was proven to be suitable for the prediction of blood pressure in SHRs.

## 4. Discussion

Clinical studies have shown that men have a higher prevalence of hypertension compared with premenopausal women [37,38]. In this study, we found that although blood pressure increased gradually from normotensive range values to hypertensive values, male SHRs had higher SBP and DBP than females. This result was consistent with the findings of Ashton and Rechelhoff, that is, gender differences in the progression of hypertension [37,39]. Previous studies have shown that hypertension and body weight are closely related. Weight gain is not only a concomitant event in hypertension; body weight also induces hypertension, especially among people with obesity [40,41,42]. Consistent results were found in our research, that is, the body weights of SHRs continuously increased with increased blood pressure [43,44].

Nutritional intervention and disease would lead to changes in VOCs in metabolite odor information of exhaled breath, urine, feces, sweat, and blood. A significant difference was found in the fecal odor between patients with colorectal cancer and healthy controls [45]. The antioxidant effects of goat and bovine whey powder were evaluated noninvasively with E-nose sensors using fecal odor [34,46]. MHC congenic mouse strains, MHC class I mutant mice, HLA-A2 transgenic mice strains, and gender difference with the same genotype were distinguished using urine or serum odor [47]. Therefore, the detection of odor types using an E-nose can be carried out objectively and rapidly for mass detection of mice with different genotypes independent of behavioral studies. In this study, the fecal odor of SHRs with different blood pressure statuses varied. Combining with PCA and CDA, SHRs with different blood pressure levels could be distinguished using fecal odor information, and the rising of blood pressure was monitored. MLP analysis was proven effective in the prediction of SBP and DBP in SHRs (0.93r0.97). These results were similar to the process monitor and intervention volatile organic assessment of goat whey powder and bovine whey powder, in which the aging status of mice was assessed by fecal odor response to an E-nose [34,46]. Therefore, we established a fast and effective non-invasive method of blood monitoring for rats using an E-nose, without disturbing the animals. The rate of accuracy (98.46%) of the MLP prediction model is considered to be acceptable. The E-nose’s fecal detection only took 80 s, as mentioned in the Results section of the manuscript, and the whole process took less than 10 min. In this case, monitoring the blood pressure level for a large number of rats was more effective than traditional non-invasive methods such as Doppler sphygmomanometry, which takes approximately 30 min for one rat. In previous studies, stress in early life altered behavior, immunity, and microbiota in rats [48]. In this study, the fecal detection capability of the E-nose did not require animal manipulation; thus, stress in rats was not stimulated, and the behavior was not altered.

## 5. Conclusions

Compared with traditional non-invasive blood pressure measurement methods, tracking blood pressure changes based on odor information of volatile metabolites in feces by E-nose is a simple process. This method is highly responsive, reproducible, and suitable for long-term and dynamic observation, which has shown great application potential as an alternative to invasive blood pressure monitoring for experimental animals.

## Figures and Tables

**Table 1:** Clinical Practice Guide for Hypertension.

BP Category		SBP/mmHg	DBP/mmHg	Age of SHRs (Weeks)	
				Male	Female
Normal		≤130	≤80	6th	6th
Hypertension	Stage 1	130~139	80~89		9th
	Stage 2	≥140	≥90	9th–18th	12th–18th

**Table 2:** Effect of different weeks on response of fecal E-nose sensor in SHRs.

Gender	Sensor Response	Age of SHRs (Weeks)				
		6	9	12	15	18
Males	S1	0.54 ± 0.01 ^d^	0.68 ± 0.01 ^c^	0.78 ± 0.01 ^a^	0.73 ± 0.01 ^b^	0.65 ± 0.01 ^c^
	S2	0.83 ± 0.01 ^c^	0.99 ± 0.03 ^c^	1.37 ± 0.09 ^b^	1.69 ± 0.19 ^a^	1.42 ± 0.15 ^a^
	S3	0.80 ± 0.01 ^d^	0.91 ± 0.01 ^c^	0.96 ± 0.01 ^a^	0.93 ± 0.01 ^b^	0.91 ± 0.01 ^c^
	S4	1.04 ± 0.01 ^d^	1.07 ± 0.01 ^c^	1.07 ± 0.01 ^c^	1.13 ± 0.01 ^b^	1.19 ± 0.01 ^a^
	S5	0.86 ± 0.01 ^d^	0.93 ± 0.01 ^b^	0.96 ± 0.01 ^a^	0.93 ± 0.01 ^b^	0.91 ± 0.01 ^c^
	S6	5.09 ± 0.22 ^a^	3.38 ± 0.11 ^b^	2.54 ± 0.08 ^d^	3.64 ± 0.06 ^b^	5.15 ± 0.18 ^a^
	S7	2.36 ± 0.08 ^c^	3.61 ± 0.26 ^b^	3.87 ± 0.34 ^b^	3.88 ± 0.30 ^b^	6.72 ± 0.27 ^a^
	S8	2.21 ± 0.06 ^b^	1.88 ± 0.04 ^c^	1.45 ± 0.03 ^d^	1.92 ± 0.02 ^c^	2.42 ± 0.05 ^a^
	S9	2.24 ± 0.07 ^c^	2.22 ± 0.06 ^c^	2.20 ± 0.08 ^c^	2.72 ± 0.14 ^b^	3.26 ± 0.16 ^a^
	S10	2.11 ± 0.05 ^a^	1.42 ± 0.02 ^c^	1.25 ± 0.01 ^d^	1.62 ± 0.01 ^c^	1.86 ± 0.03 ^b^
Females	S1	0.49 ± 0.06 ^b^	0.81 ± 0.07 ^a^	0.79 ± 0.10 ^a^	0.79 ± 0.07 ^a^	0.80 ± 0.07 ^a^
	S2	0.84 ± 0.08 ^c^	1.16 ± 0.27 ^c^	1.04 ± 0.15 ^c^	1.70 ± 0.67 ^b^	2.78 ± 1.41 ^a^
	S3	0.75 ± 0.05 ^b^	0.96 ± 0.03 ^a^	0.95 ± 0.04 ^a^	0.95 ± 0.02 ^a^	0.96 ± 0.02 ^a^
	S4	1.07 ± 0.04 ^b^	1.02 ± 0.03 ^b^	1.08 ± 0.07 ^b^	1.10 ± 0.37 ^b^	1.25 ± 0.38 ^a^
	S5	0.81 ± 0.05 ^b^	0.96 ± 0.02 ^a^	0.96 ± 0.03 ^a^	0.95 ± 0.02 ^a^	0.95 ± 0.02 ^a^
	S6	7.41 ± 1.70 ^a^	2.10 ± 0.53 ^c^	2.47 ± 1.13 ^bc^	2.70 ± 0.71 ^bc^	3.12 ± 1.24 ^b^
	S7	2.80 ± 0.36 ^b^	2.91 ± 0.54 ^b^	3.01 ± 0.79 ^b^	5.38 ± 2.51 ^a^	6.32 ± 4.55 ^a^
	S8	2.62 ± 0.38 ^a^	1.46 ± 0.26 ^b^	1.58 ± 0.41 ^b^	1.51 ± 0.28 ^b^	1.54 ± 0.26 ^b^
	S9	2.69 ± 0.46 ^a^	1.90 ± 0.16 ^b^	1.94 ± 0.31 ^b^	2.46 ± 0.51 ^a^	2.58 ± 1.00 ^a^
	S10	2.74 ± 0.39 ^a^	1.24 ± 0.13 ^c^	1.28 ± 0.22 ^c^	1.34 ± 0.13 ^bc^	1.42 ± 0.13 ^b^

Note: The data with different capital letters in the same rows show significant difference at 0.05 level, and the data with different capital letters in the same columns show significant difference at 0.05 level.

**Table 3:** Results of the confusion matrix for discriminating the blood pressure status.

Gender	BP Status	Males N	Males H	Females N	Females H	N	H	Correct Classification Rate
Male	N	26	9	0	0			60.49%
	H	7	78	57	1			
Female	N	0	12	27	1			
	H	1	2	55	91			
No distinction of gender	N					75	0	100%
	H					0	292	

Note: N: normotension, H: hypertension, BP status: blood pressure status.

**Table 4:** MLP grid performance parameters.

Input Data	Gender	BP Status	Accuracy	Precision	Sensitivity	Specificity	AUC
E-nose responses	Male	N	95.36%	74.29%	76.47%	97.30%	87.03%
		H	76.02%	54.55%	77.23%	75.56%	76.40%
	Female	N	65.94%	87.18%	19.42%	94.30%	56.86%
		H	83.65%	61.07%	97.85%	78.83%	88.34%
	No distinction of gender	N	100%	100%	100%	100%	100%
		H	100%	100%	100%	100%	100%

Note: N: normotension, H: hypertension, BP status: blood pressure status.

**Table 5:** Quantitative prediction of blood pressure in SHRs based on MLR, PLS and MLP.

Prediction Methods	Group	BP	Training Set		Test Set	
			*R* ^2^	RMES_C_	*R* ^2^	RMES_P_
MLR	Male	SBP	0.9382	8.1777	0.9509	7.4234
		DBP	0.9279	5.6242	0.9248	5.9631
	Female	SBP	0.9421	9.4059	0.9100	9.4539
		DBP	0.8357	7.1069	0.8036	8.1333
	No distinction	SBP	0.8252	12.3431	0.7779	12.4202
		DBP	0.7983	10.1148	0.7392	10.2085
PLS	Male	SBP	0.9371	8.2486	0.9551	7.0407
		DBP	0.9276	5.6451	0.9284	5.7763
	Female	SBP	0.9231	8.4741	0.9192	8.7428
		DBP	0.8326	7.1899	0.8196	7.4555
	No distinction	SBP	0.8240	14.2090	0.7848	15.5468
		DBP	0.7947	9.1503	0.7552	10.0995
MLP	Male	SBP	0.9924	2.8988	0.9926	2.9913
		DBP	0.9664	3.8458	0.9494	4.7290
	Female	SBP	0.9358	7.8121	0.8892	10.1845
		DBP	0.9103	5.2558	0.6987	9.8762
	No distinction	SBP	0.9161	9.8250	0.9325	8.9503
		DBP	0.9213	5.6714	0.8765	7.1162

## Data Availability

The data presented in this study are available in this article.

## Abbreviations

E-nose	Electronic nose
VOCs	Volatile organic compounds
SHR	Spontaneous hypertension rat
BP	Blood pressure
SBP	Systolic blood pressure
DBP	Diastolic blood pressure
PCA	Principal component analysis
CDA	Canonical discriminant analysis
MLR	Multiple linear regression
MLP	Multilayer perception neural networks
*R* ^2^	Coefficients of determination
RMSE	Root mean square error
